# MFG-E8 Reduces Aortic Intimal Proliferation in a Murine Model of Transplant Vasculopathy

**DOI:** 10.3390/ijms23084094

**Published:** 2022-04-07

**Authors:** Benoit Brilland, Patrick Laplante, Pamela Thebault, Karen Geoffroy, Marie-Joëlle Brissette, Mathieu Latour, Michaël Chassé, Shijie Qi, Marie-Josée Hébert, Héloïse Cardinal, Jean-François Cailhier

**Affiliations:** 1 Service de Néphrologie-Dialyse-Transplantation, CHU d’Angers, F-49000 Angers, France; benoit.brilland@chu-angers.fr; 2 University of Angers, Université de Nantes, CHU Angers, INSERM, CRCINA, SFR ICAT, F-49000 Angers, France; 3 Centre de Recherche du Centre Hospitalier de l’Université de Montréal (CRCHUM), Montreal, QC H2X 0A9, Canada; laplantepat@videotron.ca (P.L.); pamela.thebault@gmail.com (P.T.); ka.geoffroy@gmail.com (K.G.); marie-joelle.brissette@umontreal.ca (M.-J.B.); michael.chasse@umontreal.ca (M.C.); shijie.qi.chum@gmail.com (S.Q.); marie-josee.hebert.chum@ssss.gouv.qc.ca (M.-J.H.); heloise.cardinal.chum@ssss.gouv.qc.ca (H.C.); 4 Institut du Cancer de Montréal, Montréal, QC H2X 0A9, Canada; 5 Department of Pathology, Centre Hospitalier de l’Université de Montréal, Montreal, QC H2X 3J4, Canada; mathieu.latour.chum@ssss.gouv.qc.ca; 6 Department of Medicine, Critical Care Division, Centre Hospitalier de l’Université de Montréal, Montreal, QC H2X 3J4, Canada; 7 Canadian National Transplant Research Program, Edmonton, AB T6G 2E1, Canada; 8 Department of Medicine, Renal Division, Centre Hospitalier de l’Université de Montréal, Montreal, QC H2X 3J4, Canada

**Keywords:** MFG-E8, macrophage reprogramming, T-cell activation, aortic transplantation, transplant vasculopathy, murine model

## Abstract

Transplant vasculopathy is characterized by endothelial apoptosis, which modulates the local microenvironment. Milk fat globule epidermal growth factor 8 (MFG-E8), which is released by apoptotic endothelial cells, limits tissue damage and inflammation by promoting anti-inflammatory macrophages. We aimed to study its role in transplant vasculopathy using the murine aortic allotransplantation model. BALB/c mice were transplanted with fully mismatched aortic transplants from MFG-E8 knockout (KO) or wild type (WT) C57BL/6J mice. Thereafter, mice received MFG-E8 (or vehicle) injections for 9 weeks prior to histopathological analysis of allografts for intimal proliferation (hematoxylin and eosin staining) and leukocyte infiltration assessment (immunofluorescence). Phenotypes of blood leukocytes and humoral responses were also evaluated (flow cytometry and ELISA). Mice receiving MFG-E8 KO aortas without MFG-E8 injections had the most severe intimal proliferation (*p* < 0.001). Administration of MFG-E8 decreased intimal proliferation, especially in mice receiving MFG-E8 KO aortas. Administration of MFG-E8 also increased the proportion of anti-inflammatory macrophages among graft-infiltrating macrophages (*p* = 0.003) and decreased systemic CD4^+^ and CD8^+^ T-cell activation (*p* < 0.001). An increase in regulatory T cells occurred in both groups of mice receiving WT aortas (*p* < 0.01). Thus, the analarmin MFG-E8 appears to be an important protein for reducing intimal proliferation in this murine model of transplant vasculopathy. MFG-E8 effects are associated with intra-allograft macrophage reprogramming and systemic T-cell activation dampening.

## 1. Introduction

Transplant vasculopathy (TV) is a major cause of long-term allograft dysfunction in renal and heart transplantation [1,2]. TV is a progressive vaso-occlusive disease that leads to fibroproliferative endarteritis, characterized by the intimal accumulation of mononuclear cells, vascular smooth muscle cells (VSMCs), myofibroblasts and connective tissue. Acute vascular injury (e.g., ischemia–reperfusion injury) and ongoing alloimmune insults (e.g., delayed graft function) contribute to TV pathogenesis [2,3,4]. TV is characterized by increased endothelial apoptosis, leading to a decrease in apoptosis of VSMCs and fibroblasts, resulting in the formation of a neointima [2,5,6]. Apoptotic endothelial cells release mediators that profoundly influence the local microenvironment [2,7]. While some mediators promote inflammation by modifying the functions of resident or infiltrating immune cells (including macrophages), others allow for the subsequent remodeling and repair of the injured tissue by acting upon neighboring cells such as VSMCs and fibroblasts [8]. Those that mediate pro-inflammatory responses are termed alarmins [9], whereas others released by apoptotic endothelial cells, such as milk fat globule epidermal growth factor 8 (MFG-E8), dampen inflammation and create a microenvironment that promotes repair [10]. MFG-E8 is also known to attenuate danger signals, and thus is also referred to as an analarmin [11].

MFG-E8, also known as lactadherin, is a membrane glycoprotein expressed by many cell types including macrophages, dendritic cells and renal resident cells. MFG-E8 expression fluctuates depending on the tissue microenvironment (i.e., its health state). Our group has shown that caspase-3-dependent endothelial apoptosis promotes the release of MFG-E8 into the extracellular milieu [10]. MFG-E8 plays a major role in the phagocytosis of apoptotic cells by opsonizing their externalized phosphatidylserine, allowing them to be recognized by integrins (αvβ3, αvβ5) on the surface of macrophages [12]. Tissue MFG-E8 expression can vary considerably between pathologic conditions (i.e., inflammatory, autoimmune, infectious or malignant) [12]. We, and others, have demonstrated the anti-inflammatory effects of MFG-E8 in various murine models of acute injury [11,13,14,15,16]. We showed that MFG-E8-mediated anti-inflammatory effects are largely mediated by macrophages responding to the injury. First, MFG-E8 enhances the phagocytic clearance of apoptotic cells and thereby limits sterile inflammation [17,18]. Second, MFG-E8 contributes to the phenotype switch of macrophages from pro-inflammatory M1 to anti-inflammatory M2 macrophages, independent of apoptotic cell engulfment [10,11,13]. In addition, MFG-E8 also attenuates the local recruitment of neutrophils [19,20].

The role of MFG-E8 in solid organ transplantation, more specifically in TV, is poorly defined. We hypothesized that MFG-E8 would attenuate TV by promoting anti-inflammatory macrophages, thus dampening alloimmune responses. Using an allogeneic abdominal aortic transplant model widely used to study TV, we show that the analarmin MFG-E8 reduced aortic intimal proliferation in association with M2 macrophage reprogramming and T-cell activation dampening.

## 2. Materials and Methods

### 2.1. Transplant Vasculopathy Model and Injection of Murine Recombinant MFG-E8

**Figure 1:**
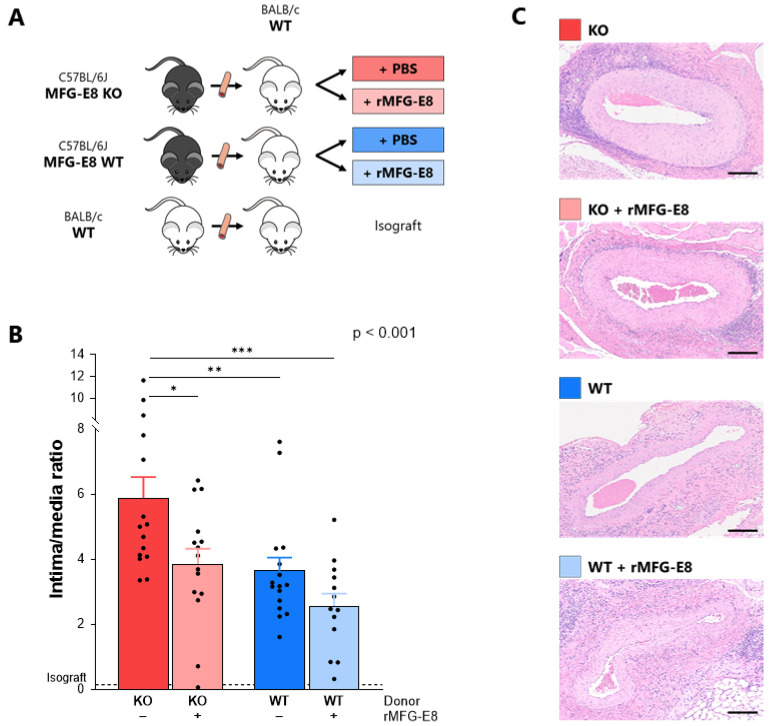
Effect of MFG-E8 on intimal proliferation in a murine model of transplant vasculopathy. (**A**) Murine aortic transplant vasculopathy model. Aorta transplantation procedures were performed between C57BL/6J and BALB/c mice. Mice subsequently received either rMFG-E8 or vehicle (PBS). (**B**) Intimal proliferation (intima/media ratio) within the grafted aorta, assessed by hematoxylin and eosin after 9 weeks. *p*-Value (upper right corner) indicates the overall ANOVA result. (**C**) Representative section of intimal proliferation in each group. Bars represent 200 µm. * *p* < 0.05, ** *p* < 0.01, *** *p* < 0.001.

The TV model was performed as previously described [21,22]: Aortas from MFG-E8 KO or WT C57BL/6J female mice were orthotopically transplanted into WT BALB/c male mice (Figure 1A). Isograft transplantations between BALB/c mice were performed as controls. Each group subsequently received either murine recombinant MFG-E8 (rMFG-E8) (R&D Systems, Minneapolis, MN, USA; 30 μg/kg in 100 μL of vehicle) or vehicle (PBS), intra-peritoneally, on day 0 and then twice weekly. Mice were 8- to 9-weeks-old, did not receive any immunosuppressive therapies and were monitored for 9 weeks (n = total of 16–17 mice per group, 4 isografts, 4 independent experiments) or 4 weeks (n = 5–6 mice per group, 1 isograft, 1 experiment). C57BL/6J and BALB/c mice were bought from Jackson Laboratory (Bar Harbor, ME, USA) and Charles River (Wilmington, MA, USA), respectively. MFG-E8 KO mice of the same C57BL/6J strain were bred and genotyped in our animal facility. Studies were approved by our institutional Animal Care Committee (Comité institutionnel de protection des animaux, CRCHUM).

### 2.2. Morphometric and Immunofluorescence Analysis of Aortic Grafts

Transplanted and adjacent native aortas were harvested and fixed in 10% neutral buffered formalin. After paraffin embedding, samples were cut into 4 µm slices and stained with hematoxylin and eosin. Intimal and media area grafts were blindly outlined and quantified using a digital image analysis program (ImageJ 1.50i, National Institutes of Health). Allograft intimal proliferation was evaluated with the intima/media ratio (ratio between intima and media areas) to quantify vascular rejection and TV [21].

Immunofluorescence was performed to study macrophages, M2 macrophages, CD4^+^ T cells and CD8^+^ T-cell infiltration (represented by Mac-2^+^, Mac-2^+^CD206^+^, CD4^+^ and CD8^+^ cells, respectively; the antibodies used are described in Supplementary Table S1) with the following protocol: After deparaffinization in three successive 5 min baths of xylene and rehydration in an ethanol gradient, antigen retrieval of the aorta was achieved with EDTA buffer (1 mM + 0.05% Tween 20 adjusted to pH 8.0) for 20 min at boiling temperature. Tissues were washed, here and after every subsequent step, permeabilized with 0.25% Triton X-100 in PBS for 30 min, blocked with a blocking buffer (Life Technologies, CA, USA) for 1 h, incubated at 4 °C overnight with primary antibody, incubated for 1 h with secondary antibody the following day and finally counterstained using ProLong Gold Antifade Reagent with DAPI (Molecular Probes, Sunnyvale, CA, USA). Substitution of the primary antibodies with blocking buffer was used as a negative control. A Zeiss Observer Z1 fluorescent microscope (Program AxioVision 4.8, Zeiss, Germany) was used to view the slides. The examiner was blinded to the experimental conditions and took pictures at three random areas for every section. Leukocyte infiltration was manually assessed by counting positive cells in each area.

### 2.3. Flow Cytometric Analyses of Lymphocytes

Blood was collected from each mouse every week. After plasma isolation and red blood cell lysis, leukocytes were incubated (15 min, 4 °C) with FC Block (anti-CD16/CD32, eBioscience, San Diego, CA, USA) to avoid non-specific binding. After washing, cells were stained for 30 min with extracellular antibodies (anti-CD3, -CD4, -CD8, -CD25, -CD44, -CD62L antibodies, Supplementary Table S2). After fixation and permeabilization (FoxP3/Transcription factor staining buffer set, eBioscience), cells were incubated for 30 min with intracellular antibodies to identify regulatory T cells (anti-FoxP3 antibodies, Supplementary Table S2). Expression of the various cell markers was determined using a flow cytometer (BD LSRFortessa, BD Biosciences, Franklin Lakes, NJ, USA) and analyzed using FlowJo v10.3 (FlowJo, LLC, Ashland, OR, USA). The gating strategy is described in Supplementary Figure S1.

### 2.4. Assessment of Humoral Responses and Anti-Donor Antibodies

Total IgG levels were assessed by ELISA (Affymetrix, Santa Clara, CA, USA) according to the manufacturer’s instructions. Murine anti-donor antibodies (ADAs) were indirectly measured by flow cytometry as previously described [21]. Briefly, neat sera from BALB/c transplanted mice were incubated with 1 × 10^6^ C57BL/6J splenocytes (30 min, 4 °C). Samples were then washed three times and stained with phycoerythrin (PE) goat anti-mouse IgG (Biolegend, San Diego, CA, USA) and AF488 anti-mouse CD3 (BD Biosciences) in FACS buffer for 30 min at 4 °C in the dark. Cells were then passed through a flow cytometer (BD LSRFortessa, BD Biosciences) and analyzed using FlowJo v10.3 (FlowJo, LLC). The gating strategy is described in Supplementary Figure S2.

### 2.5. Statistical Analyses

Mice data are expressed as means ± SEMs. Biological and histological data were compared using ANOVA and Tukey post-test correction or Student’s *t*-test and Sidak–Bonferroni post-test correction. Statistical analyses were performed using Prism 6 (GrahPad, San Diego, CA, USA).

## 3. Results

### 3.1. Absence of MFG-E8 Promoted Intimal Proliferation in the Transplanted Aorta

At week 9, intimal proliferation was significantly higher in mice receiving MFG-E8 KO aortas (ratio = 5.88 ± 0.65) when compared to mice receiving WT aortas (3.64 ± 0.41, *p* < 0.01). The addition of rMFG-E8 significantly decreased intimal proliferation in mice receiving MFG-E8 KO aortas (3.85 ± 0.47 vs. 5.88 ± 0.65, *p* < 0.05). Although not significant, the addition of rMFG-E8 also decreased intimal proliferation in mice receiving MFG-E8 WT aortas. Lastly, mice receiving WT aortas and rMFG-E8 had the lowest intimal proliferation (2.56 ± 0.38, *p* < 0.001 vs. MFG-E8 KO group) (Figure 1B,C). We found the same results in allografts harvested at 4 weeks (Supplementary Figure S3). Whether assessed at 4 or 9 weeks, intimal proliferation was almost non-existent in the isograft control group, suggesting that alloimmune mechanisms were responsible for this intimal thickening, which is characteristic of TV. Based on this observation, this group was excluded from further analyses.

### 3.2. MFG-E8 Increased the Proportion of M2 Anti-Inflammatory Macrophages among Infiltrative Macrophages

**Figure 2:**
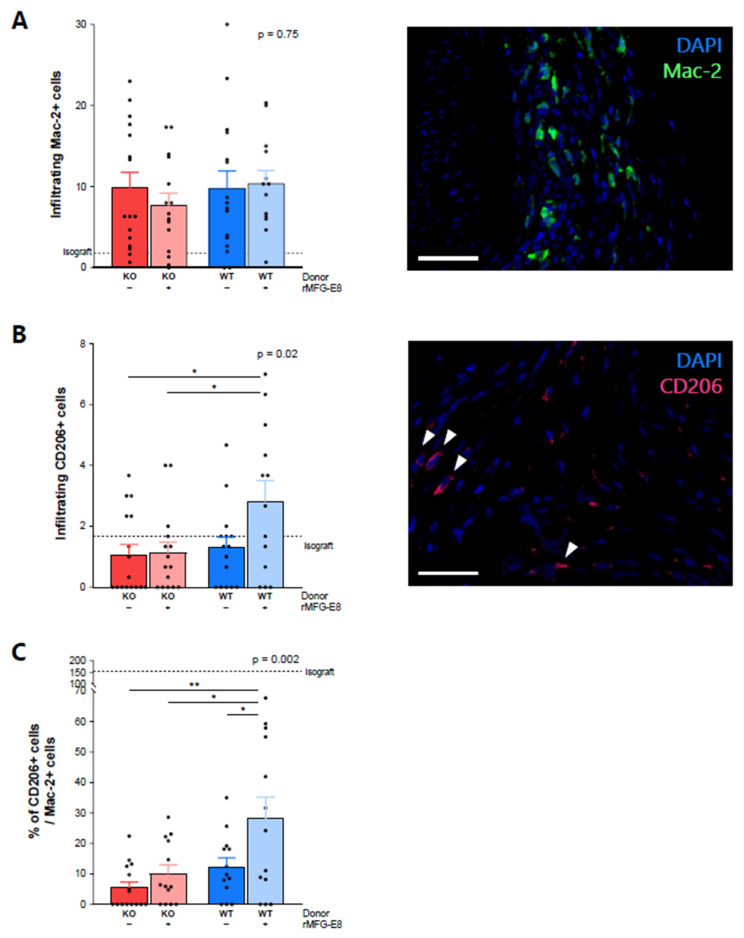
Effect of MFG-E8 on macrophage allograft infiltration. (**A**) Macrophage infiltration within the transplanted aorta, assessed by immunofluorescence positivity of Mac-2^+^ cells staining (in green). (**B**) CD206^+^ cell infiltration (pink staining) within the transplanted aorta. (**C**) Ratio of CD206^+^ cells/Mac-2^+^ cells. Values represent the average number of positive cells in three random areas. All data were assessed 9 weeks after transplantation. White bars represent 50 µm. *p*-Value (upper right corner) indicates the overall ANOVA result. * *p* < 0.05, ** *p* < 0.01.

At week 9, there was no difference between groups in the number of macrophages infiltrating the grafted aortas (Figure 2A, Mac-2^+^ cells, *p* = 0.75). However, they were more numerous than in the isograft control group. There was a significant increase in CD206^+^ (an M2 macrophage marker) infiltrating cells in mice receiving WT aortas and rMFG-E8, especially when compared to mice receiving KO aortas (Figure 2B, *p* = 0.02). The ratio of infiltrating CD206^+^ cells/infiltrating Mac-2^+^ cells was significantly higher in mice receiving WT aortas and rMFG-E8 (Figure 2C, *p* = 0.002) than in the other groups. These results suggest that the presence of MFG-E8 can promote an anti-inflammatory phenotype in infiltrating macrophages in the context of transplantation.

### 3.3. MFG-E8 Administration and T-Cell Infiltration into Allografts

**Figure 3:**
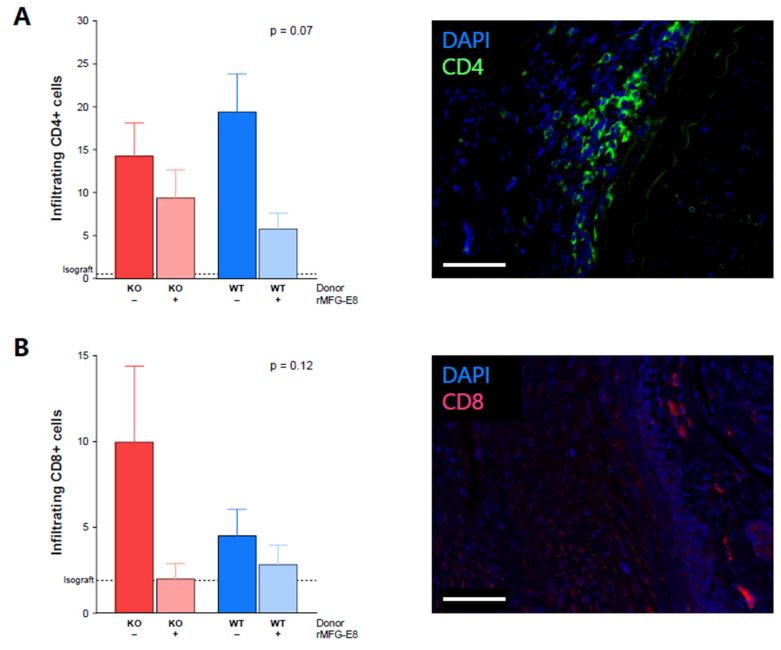
Effect of MFG-E8 on T-cell allograft infiltration. (**A**) CD4^+^ cell infiltration within the transplanted aorta, assessed by immunofluorescence (green staining). (**B**) CD8^+^ cell infiltration within the transplanted aorta, assessed by immunofluorescence (pink staining). Values represent the average number of positive cells in three random areas. All data were assessed 9 weeks after transplantation. White bars represent 50 µm. *p*-Value (upper right corner) indicates the overall ANOVA result.

Because of their importance in TV [23], we evaluated T-cell infiltration within the allografts. At week 9, we found a trend of decreased infiltration of CD4^+^ cells in mice receiving rMFG-E8 (Figure 3A, *p* = 0.07). While not significant, we also observed an increased infiltration of CD8^+^ cells in mice receiving a KO aorta and no rMFG-E8 (Figure 3B, *p* = 0.12). In both cases, the isograft control group had few infiltrating T cells (Figure 3A,B).

### 3.4. MFG-E8 Dampened Systemic T-Cell Activation

**Figure 4:**
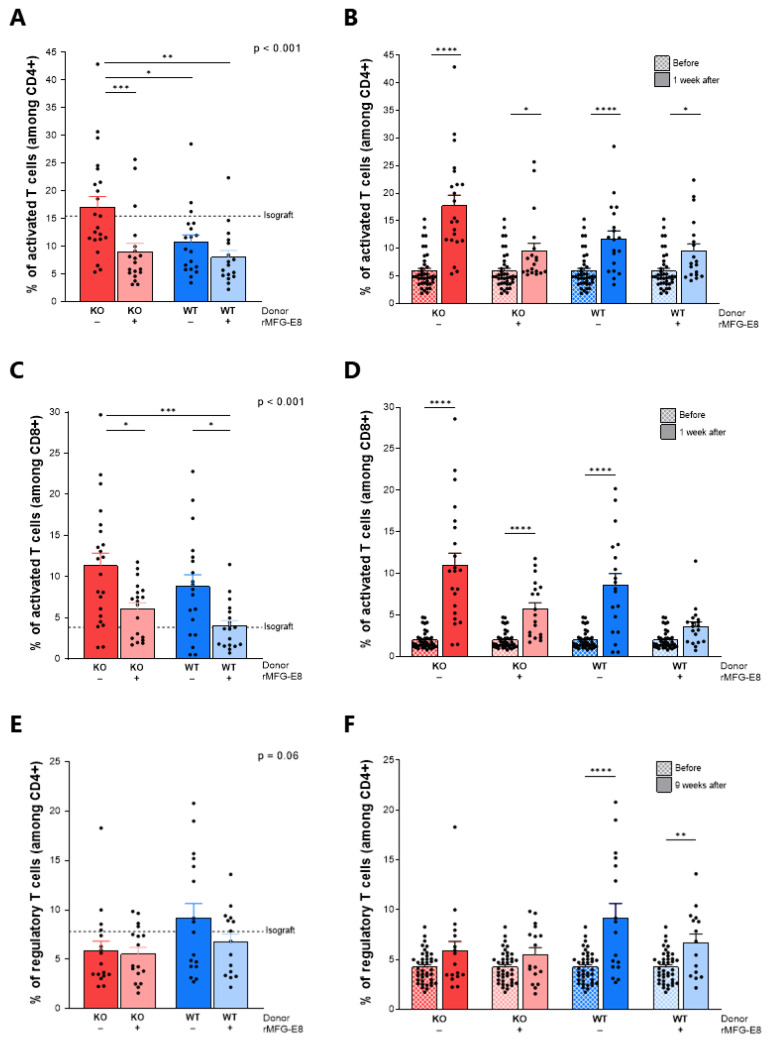
Effect of MFG-E8 on systemic T-cell activation. Systemic T-cell activation was assessed by flow cytometry (gating strategies are shown in Supplementary Figure S1). Activated CD4^+^ CD44^+^ T cells 1 week after transplantation (**A**) and comparison with levels before transplantation (**B**). Activated CD8^+^ CD44^+^ T cells 1 week after transplantation (**C**) and comparison with levels before transplantation (**D**). CD25^+^FoxP3^+^ regulatory T cells 9 weeks after transplantation (**C**) and comparison with levels before transplantation (**D**). In figures (**A**,**C**,**E**), *p*-values (upper right corner) indicate the overall ANOVA result. In figures (**B**,**D**,**F**), hatched bars represent pre-transplant data. Solid bars represent post-transplant data. * *p* < 0.05, ** *p* < 0.01, *** *p* < 0.001, **** *p* < 0.0001.

Next, we evaluated the frequency of circulating activated CD4^+^ T cells, activated CD8^+^ T cells and regulatory CD4^+^ T cells in the allograft recipients (Supplementary Figure S1). One week after transplantation, we observed a higher frequency of activated CD4^+^ T cells (percentage of CD44^+^ cells among CD3^+^CD4^+^ cells) in mice receiving KO aortas and no rMFG-E8 compared to the other groups (Figure 4A). Moreover, if an increase in CD4 activation was noted in every group when compared to pre-transplant levels, this increase was more pronounced in mice that did not receive rMFG-E8 compared to those that did (Figure 4B, *p* < 0.0001).

The administration of rMFG-E8 was also associated with a decrease in the frequency of activated CD8^+^ T cells (percentage of CD44^+^ cells among CD3^+^CD8^+^ cells) in both KO and WT aorta-transplanted mice (Figure 4C, *p* < 0.05). Furthermore, there was a significant increase in CD8 activation in each group, except for mice receiving a WT aorta and rMFG-E8 (Figure 4D, *p* < 0.0001).

While no differences in Tregs (percentage of CD25^+^FoxP3^+^ cells among CD3^+^CD4^+^ cells) were noted immediately after transplantation between any of the groups, the percentage of Tregs after 9 weeks tended to be lower in mice receiving a KO aorta compared to those receiving WT aortas (Figure 4E, *p* = 0.06). Moreover, an increase in Tregs was observed only in mice receiving a WT aorta (Figure 4F, *p* < 0.01).

### 3.5. MFG-E8 Did Not Significantly Impact Humoral Responses

We found that, after 9 weeks, MFG-E8-naïve mice (receiving a KO aorta and no injection of rMFG-E8) tended to produce more IgG (Supplementary Figure S4A, *p* = 0.10). Four weeks after transplantation, ADAs were non-significantly higher in mice receiving KO aorta and no rMFG-E8 (Supplementary Figure S4B, *p* = 0.35), while they became abundant in each group after 9 weeks (Supplementary Figure S4C, *p* = 0.33).

## 4. Discussion

In this study, we analyzed the role of MFG-E8 in the context of transplantation in a mouse model of TV. We showed that the absence of MFG-E8 in murine aortic allografts was associated with intimal proliferation. Interestingly, TV in MFG-E8 KO aortas was significantly attenuated by the administration of rMFG-E8. rMFG-E8 injections in mice receiving MFG-E8 WT aortas did not significantly affect TV, suggesting that there was a sufficient contribution of endogenous MFG-E8 to prevent TV. To determine the factors that were associated with intimal proliferation, we analyzed histopathological data, macrophage and lymphocyte phenotypes and humoral response in transplanted mice.

First, we observed that, while macrophage infiltration was not modified by endogenous MFG-E8 expression in the grafts or rMFG-E8 administration, the proportion of M2 macrophages among infiltrating macrophages was significantly increased in mice receiving WT aortas and rMFG-E8. We assume that the acquisition of this M2 phenotype by macrophages was directly influenced by MFG-E8, as shown previously by our group [11,13], and by the known effect of exogenous MFG-E8 on efferocytosis process facilitation that triggers a regulatory phenotype [24,25]. We hypothesize that the MFG-E8-induced M2 phenotype of infiltrating macrophages has regulatory properties in this setting, as demonstrated by other studies [26,27,28]. The promotion of uncontrolled M2 macrophages could potentially lead to a pro-fibrotic microenvironment by inducing tissue fibrosis and uncontrolled repair. However, increasing available MFG-E8 reduced the intensity of the pro-inflammatory response, precluding tissue damage and further uncontrolled repair, leading to myointimal proliferation. Promoting an early M2 phenotype with MFG-E8 also opposed the initial inflamed microenvironment, thereby reducing this maladaptive repair response.

This increase in anti-inflammatory macrophages may be involved in the decrease in T-cell infiltration that we observed, both of which led to the observed decrease in intimal proliferation.

Second, we observed that, immediately after transplantation, the absence of MFG-E8 was associated with a higher percentage of activated CD4^+^ and CD8^+^ T cells in peripheral blood and that the injection of rMFG-E8 decreased the frequency of activated CD4^+^ T cells. Moreover, a significant increase in Tregs was only observed in mice receiving WT aortas. Although lymphocytes do not possess an integrin receptor [17], the MFG-E8 receptor, Tan et al. recently reported that MFG-E8 can directly downregulate effector T cells while upregulating Tregs in a PKCθ-dependent manner [29]. Indirectly, MFG-E8 could have locally promoted tolerogenic antigen-presenting cells (dendritic cells, M2 macrophages) that would facilitate the generation of Tregs, thus limiting systemic pro-inflammatory T-cell responses [30,31,32].

Last, while we did not find any change in B-cell systemic activation (data not shown), we found a non-significant trend for higher ADA and total IgG levels in mice receiving KO aortas and no rMFG-E8. This suggests that B cells, which play a key role in the pathogenesis of antibody-mediated acute rejection and chronic allograft rejection [33,34], could be impacted by MFG-E8 effects, at least indirectly.

Our study encounters several limitations. First, though widely used, the accuracy of aorta transplantation in modelling TV and chronic rejection is debated [35,36,37], mostly because of the endothelial nature of the graft. Second, while interesting, immunofluorescence results are limited by the lack of statistical significance and were performed with markers that can be attributed to other cell types (CD8^+^ by dendritic cells or NK cells, and CD4^+^ by monocytes). Lastly, we only used CD206^+^ as a marker for M2 macrophages; we did not study M1 markers to complete our results.

## 5. Conclusions

To conclude, these results suggest a beneficial role for MFG-E8 in the context of transplantation. MFG-E8 appears to regulate intimal proliferation in a mouse model of chronic TV. While additional studies are needed to understand the role of MFG-E8 in modulating macrophage phenotypes and its (probably indirect) role in lymphocyte activation, our results suggest that factors regulating the expression of MFG-E8 or MFG-E8 itself could be considered potential therapeutic targets.

## Data Availability

Data available on request to the corresponding author.

## Supplementary Materials

The following supporting information can be downloaded at: https://www.mdpi.com/article/10.3390/ijms23084094/s1.

## Abbreviations

ADA	Anti-Donor Antibody
DGF	Delayed Graft Function
DSA	Donor-Specific Antibody
GFR	Glomerular Filtration Rate
IQR	Interquartile Range
KO	Knockout
MFG-E8	Milk Fat Globule Epidermal Growth Factor 8
PBS	Phosphate-Buffered Saline
PE	Phycoerythrin
rMFG-E8	Recombinant MFG-E8
Tregs	Regulatory T cells
TV	Transplant Vasculopathy
VSMCs	Vascular Smooth Muscle Cells
WT	Wild Type
