# Supplementary material for: MFG-E8 Reduces Aortic Intimal Proliferation in a Murine Model of Transplant Vasculopathy
Source: Int J Mol Sci. 2022 Apr 7;23(8):4094. doi: 10.3390/ijms23084094 (PMC9027378; doi:10.3390/ijms23084094)
Supplement: Supplementary file 1 [file ijms-23-04094-s001.zip › ijms-1644431-supplementary.pdf]

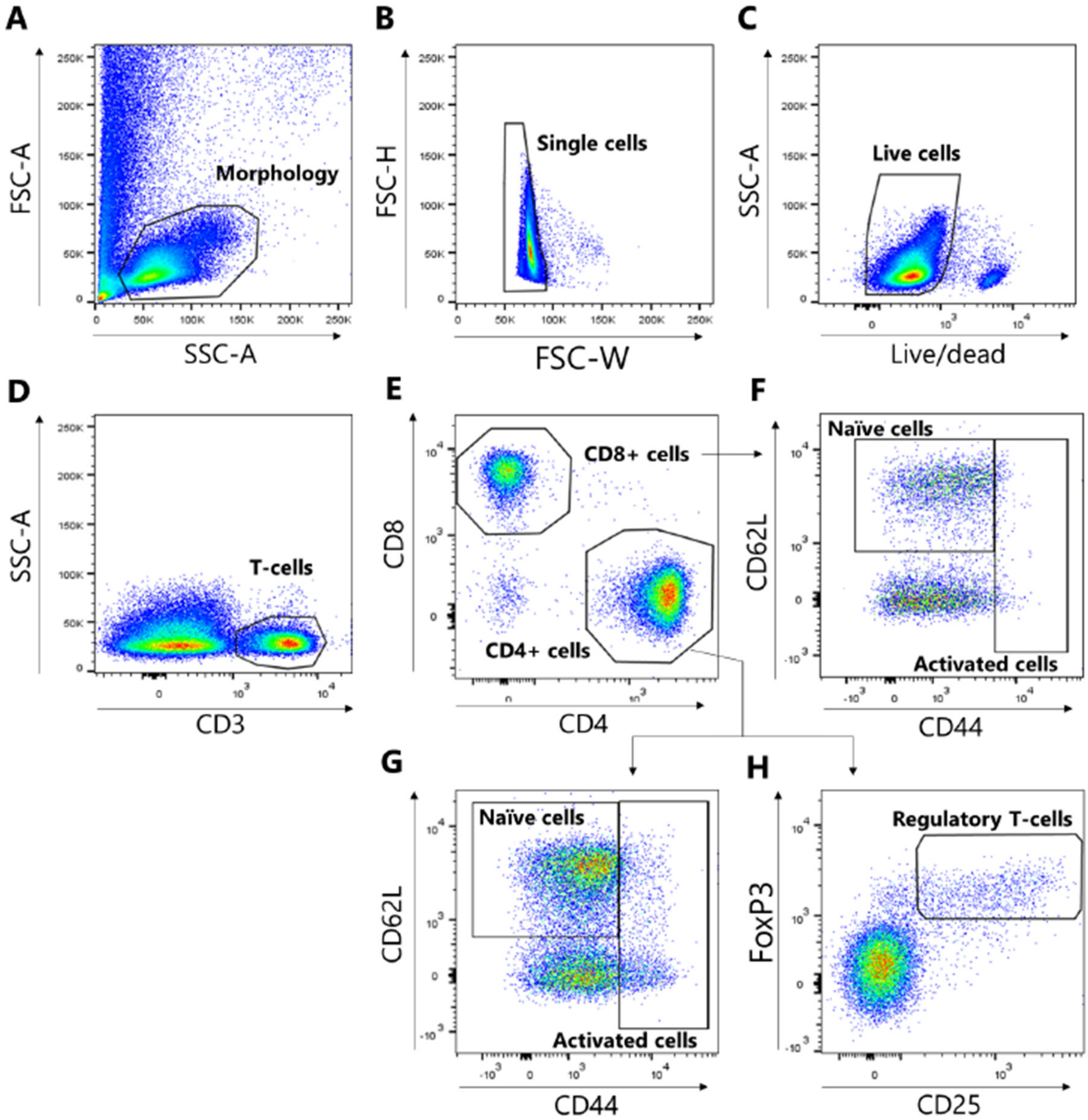

Supplementary Figure S1. Flow cytometry gating strategy to evaluate T-cell activation status. (**A**) Leukocytes were selected according to their morphology, (**B**) doublets were excluded, and (**C**) living cells were selected using a viability dye. (**D**) T cells ($CD3^+$ cells) were selected and (**E**) $CD4^+$ or $CD8^+$ cells were isolated. (**F,G**) Among them, naïve ($CD62L^+CD44^-$) or activated cells ($CD44^+$ cells) were gated. (**H**) From the $CD3^+CD4^+$ cells, $CD25^+FoxP3^+$ cells are regulatory T cells.

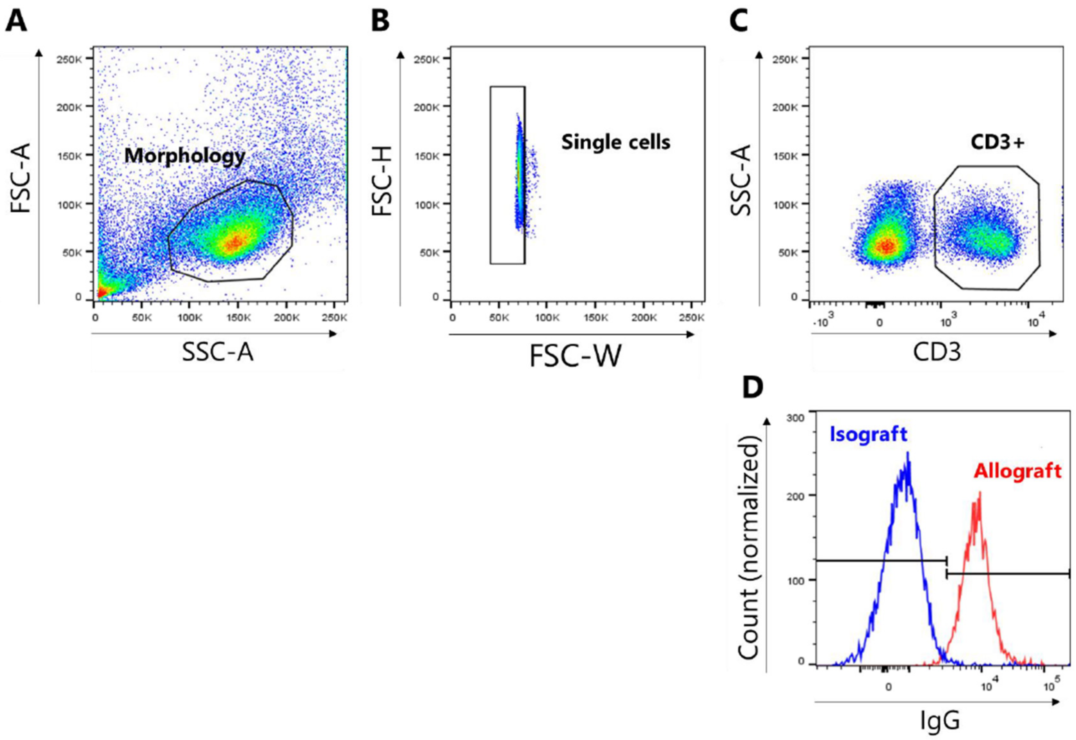

Supplementary Figure S2. Flow cytometry gating strategy to evaluate the presence of anti-donor antibodies. After selection of splenocytes (**A**) and single cells (**B**), IgG^+ cells (**D**) among $CD3^+$ cells (**C**) were considered positive for anti-donor antibodies.

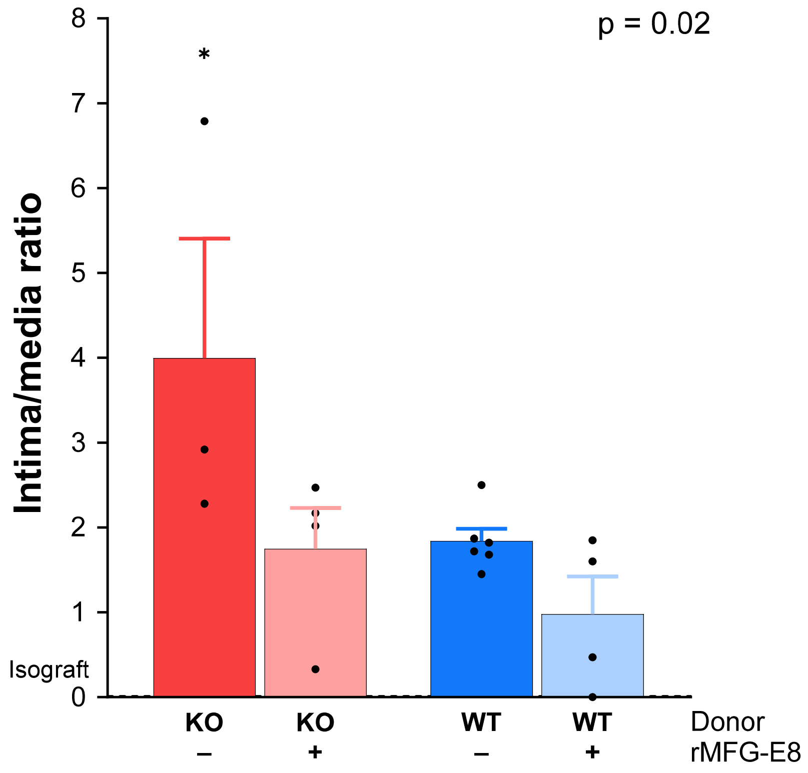

Supplementary Figure S3. Early effects of MFG-E8 on intimal proliferation. Intimal proliferation, assessed at 4 weeks following transplantation, was higher in mice receiving MFG-E8 KO aortas ($p = 0.02$). One experiment, n = 5–6 per group. * $p < 0.05$ between KO and WT + rMFG-E8 mice.

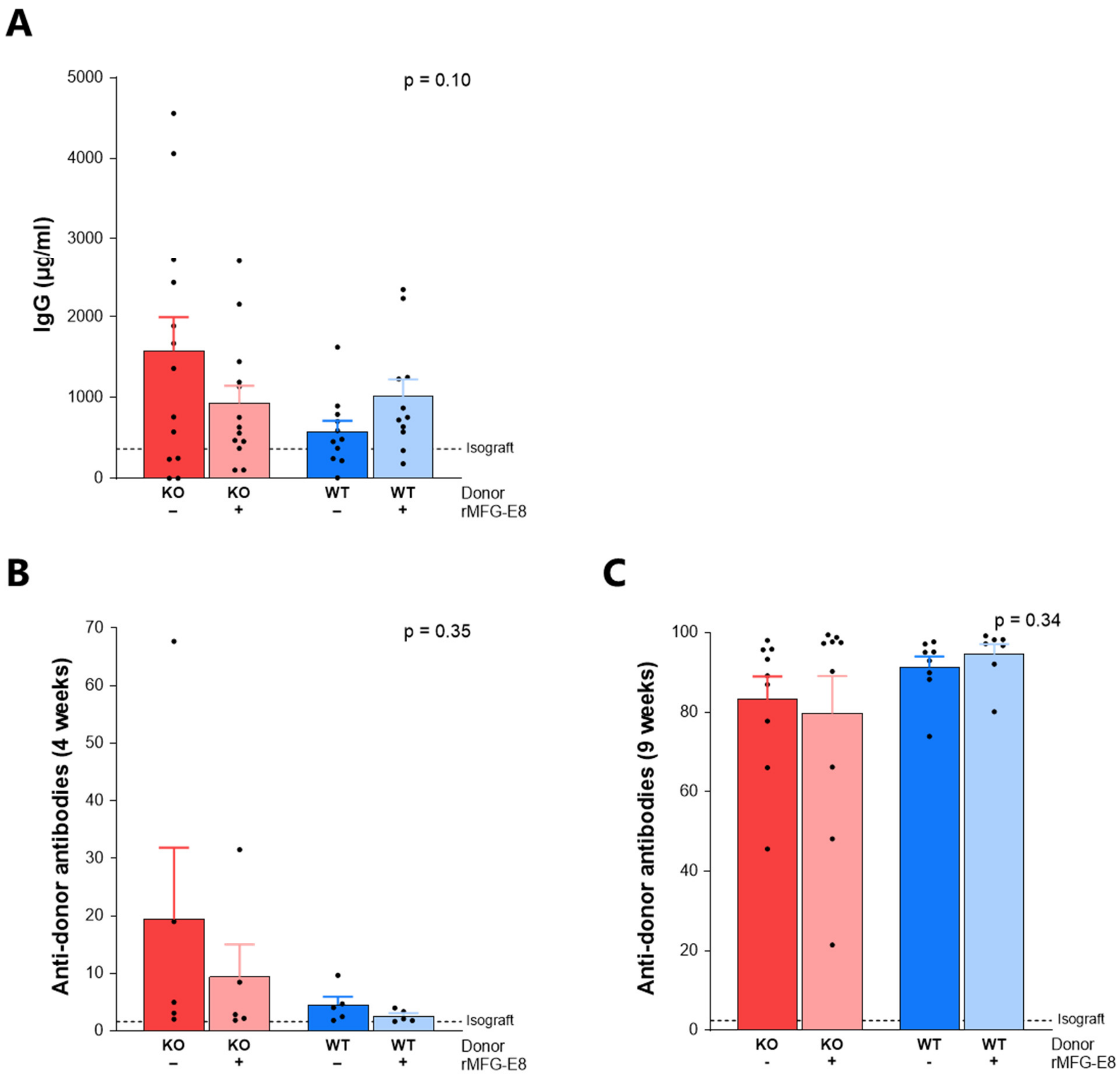

Supplementary Figure S4. Effect of MFG-E8 on cytokine and humoral response. (**A**) IgG in mice 9 weeks after transplantation**.** Anti-donor antibodies (ADAs) levels in mice 4 weeks (**B**) or 9 weeks (**C**) after transplantation. *p*-Value (upper corner) indicates overall ANOVA result.

Supplementary Table S1. Immunofluorescence primary and secondary antibodies.

	Primary antibody	Host	Provider	Secondary antibody	Provider
**T-cell infiltration**	CD4	Rat	ThermoFisher	anti-rat-AF488	Life Technologies
	CD8α	Mouse	Santa-Cruz	anti-mouse-AF594	Life Technologies
**Macrophage infiltration**	Mac-2	Rat	Biolegend	anti-rat-AF488	Life Technologies
	CD206	Rabbit	Abcam	anti-rabbit-AF647	Life Technologies

Mac-2 is a macrophage marker. CD206 is an M2 macrophage marker.

Abbreviations: n/a, not applicable; AF, Alexa Fluor; DAPI, 4',6-diamidino-2-phénylindole; CD, cluster of differentiation.

Supplementary Table S2. Flow cytometry antibodies.

Target	Fluorochrome	Clone	Provider
**CD3**	PerCP-Cy5.5	145-2C11	BD Biosciences
**CD4**	BUV395	GK1.5	BD Biosciences
**CD8**	V450	53-6.7	BD Biosciences
**CD44**	PECy7	IM7	BD Biosciences
**CD62L**	APC-R700	MEL-14	BD Biosciences
**CD25**	APC	PC61	BD Biosciences
**FoxP3**	PE	FJK-16s	eBioscience*
**Live/dead**	Aqua	n/a	ThermoFisher

Abbreviations: CD, cluster of differentiation; n/a, not applicable.

* FoxP3/Transcription factor staining buffer set, eBioscience.
